# High frequency social calls indicate food source defense in foraging Common pipistrelle bats

**DOI:** 10.1038/s41598-020-62743-z

**Published:** 2020-04-01

**Authors:** Simone Götze, Annette Denzinger, Hans-Ulrich Schnitzler

**Affiliations:** Animal Physiology, Institute for Neurobiology, University of Tübingen, Tübingen, Germany

**Keywords:** Behavioural ecology, Ecophysiology

## Abstract

Social calls have the function to coordinate the behavior of animals. In the presence of conspecifics foraging Common pipistrelle bats (*P. pipistrellus)* emitted, in addition to typical echolocation signals, two types of social calls: complex social calls and an as-of-yet undescribed, short, frequency-modulated call type with high terminal frequency, which we term “high frequency social call”. By recording the flight and acoustic behavior of free flying pairs of foraging *P. pipistrellus* with an array of four microphones we were able to determine their three-dimensional flight paths and attribute emitted calls to particular behavioral situations. Complex social calls were emitted at further inter-individual distances and at large bearing angles to conspecifics, whereas high frequency social calls were produced at significantly shorter distances and at smaller bearing angles. These calls were associated with chasings and the eviction of the intruder. We assume that the emission of both types of social calls by foraging bats reflects a two-stage-process of the occupation and defense of a food patch. Common pipistrelle bats use complex social calls to claim a food patch and switch to agonistic behaviors, including chasings and high frequency social call emissions, when they defend their foraging territory against an intruder.

## Introduction

Acoustic signaling plays an important role in animal communication^1^. Among mammals, bats exhibit a remarkable repertoire of social calls, including aggression and distress signals between conspecifics and for the defense against predators, signals in mother-infant interactions as well as advertisement calls for mating^2–5^.

In this study, we focused on the social call repertoire of foraging *Pipistrellus pipistrellus*, one of the most well-studied vespertilionids of Europe. This insect-eating bat emits signals for orientation, detection and localization of airborne prey^6,7^, and produces social calls for intraspecific and interspecific communication. Its echolocation and social signal design have been investigated and described by numerous studies, although their social call repertoire has yet to be fully described or understood^2,8–15^.

It has been established that pipistrelles use four types (A-D) of social calls^2^, which differ considerably in structural design. In contrast to types A and B, which are emitted only by stationary bats at the roost, Types C and D are produced by individuals during flight. Of these flight calls, Type C is a single-element contact signal used in mother-infant interactions, whereas Type D consists of 2–5 multi-harmonic frequency-modulated syllables, a call that is also referred to as ‘complex social call’^2,16^. Studies investigating the behavioral function of complex social calls have found two different contexts in which they are emitted – as part of courtship flightsongs for mating, and in foraging behavior^3,11,15,17^ – which is surprising given the opposing intentions (attraction and repulsion) intended by these two behaviors^2^. Complex social calls in both of these behavioral situations appear indistinguishable from their structure, although they show slight but significant differences^12^. These might be caused by individual variations, since Pfalzer and Kusch^2^ showed that individual bats can be distinguished by intraspecific differences in call duration and frequency. In fact, due to these individual signatures, bats may be able to identify and distinguish individual conspecifics^18–21^. The question remains, however, of how a bat could distinguish between incoming complex social calls that were either meant to attract (‘advertisement calls’^2,11,22^, ‘mating calls’^2^ or ‘songflight calls’^12^) as compared to those meant to repel (‘agonistic calls’)^10^. It has been suggested that various cues could serve to differentiate foraging and courtship flightsongs, such as the number of complex social calls in a series^10^, diverging pulse intervals^8,10,12^, season^23^, location^13^, and/or flight behavior^10,13^ of the emitter. Here, we challenge the differentiation between advertisement and agonistic calls, and rather hypothesize that complex social calls at all times serve as an announcement of a bat’s presence. If complex social calls are broadcasted by a bat at a foraging site, the signal should have a repelling effect on male and female conspecifics, because it signals the presence of a resident and the occupation of a food resource. The same type of calls can also act as an attracting signal on females and as a repellent signal for males during mating season, because the announcement of presence is then linked with the occupation of a mating territory. For simplicity, we chose the term ‘Type 1 call’ for complex social calls for the course of this study.

In the current study we also recorded signals from *Pipistrellus pipistrellus* which were different from the species’ echolocation signals and their known social calls. These were sweeps of short duration and high terminal frequency which were emitted in striking temporal proximity to Type 1 calls. We preliminary termed these ‘Type 2 calls’ and sought to understand the purpose of their emission.

We assumed that not only Type 1 but also Type 2 calls have a social function and tested the following hypotheses:

1. Type 2 calls are social calls and have an impact on the behavior of conspecifics.
2. Type 2 calls serve as agonistic social calls and elicit the eviction of intruders.

## Methods

### Species and study sites

We recorded free flying common pipistrelles (*Pipistrellus pipistrellus*) from May to October in 2010 and from 2013 to 2015. The recordings were conducted during 34 nights at 16 different sites in Brandenburg, East Germany, and Baden-Württemberg and Bavaria, South Germany. Two recording sites in Baden-Württemberg were illuminated by street lights; 14 sites were unlit. The illuminated recording sites were positioned underneath street lights at the periphery of residential areas, whereas the unlit sites were situated along forest edges and water resources such as lakes and rivers. Recordings started 30–50 minutes after sunset, lasting 1 hr 45 min on average. On individual nights the location was changed after 2 hours for additional recordings. We recorded a total of more than 57 hrs of audio, of which 37 hrs, 38 min were analyzed. All recording sites consisted of edge space habitats along structures and were connected to open space habitats. All recordings were conducted in accordance with relevant guidelines and regulations.

### Sound recordings

In the course of this study we successively used two horizontally-oriented T-shaped and planar microphone arrays of equal design but different sizes. Each array consisted of four microphones pointing upwards in the same direction; three microphones were positioned in a line and a fourth was fixed at a right angle to the central microphone. All outer microphones were placed at equal distance from the central microphone. The array used in 2010 consisted of four Knowles microphones (Model SPM0404UD5) with outer microphones at distances of 1 m from the central microphone. From 2013 to 2015, we used a larger array with custom-made microphones fixed at a distance of 2 m from the central microphone.

The arrays were positioned at ground level, or in the case of uneven ground, adjusted to heights of 1.2–1.6 m above ground. Each sound recording lasted 20 seconds and was amplified, digitized and stored as a .wav file using the custom-made software ‘Battery’. In 2010, the sound recordings were digitized with a sampling rate of 250 kHz with an A/D-converter of Type USB-6251 (National instruments, Texas). From 2013 to 2015 the sampling rate was increased to 400 kHz due to enhanced technical equipment (National instruments, A/D-converter of Type USB-6356).

### Data analysis

During the sampling period, we recorded a total of 2078 Type 1 calls and 952 Type 2 calls. We visualized the recorded signals as color spectrograms (FFT 512, Blackman window, dynamic range of 70 dB) using custom-made software (Selena, University of Tübingen, Germany). The spectrograms were plotted with a temporal resolution of 0.06 ms and a spectral resolution of 156.3 Hz due to auto-padding and time interpolation. The beginning and end of signals were measured in the spectrograms using the automatically applied criterion of −15 dB below highest amplitude. We measured several signal parameters, including duration, peak frequency, terminal frequency, bandwidth, and pulse interval and tested each for normal distribution using a Kolmogoroff-Smirnoff-Test (JMP 11.2.0). Terminal frequencies of echolocation calls, Type 1 and Type 2 calls of equal duration were compared by an ANOVA (JMP 11.2.0). Given that the data points for pulse intervals were not normally distributed, we chose median, and first (Q1) and third quantile (Q3) as a measurement for pulse intervals for all comparisons. We tested the impact of both call types on pulse intervals between search calls using a Wilcoxon test (JMP 11.2.0).

Signals of insufficient amplitude were still used for reconstruction of flight paths when possible. In addition, we counted Type 1 and Type 2 calls within our recordings over four years from May to October and calculated the emission rate for each call type per each 10-minute recording sequence.

### Differentiation of single flights and flights of two or more bats

Typically, echolocation calls from a single bat flying above the array produced a sequence of steady signals characterized by constant or smoothly changing amplitude and pulse intervals of approximately 90 ms; in some cases, however, an approach sequence with decreasing pulse intervals and durations was emitted, often ending in a feeding buzz. Terminal frequencies of echolocation signals have a negative correlation with sound duration, which was considered when using frequency for the identification of individuals.

Audio recordings of more than one bat flying within the recording area were characterized by irregular pulse intervals of less than 90 ms. Individual bats often differed in their individual terminal frequencies for distinct call durations, by which they could be distinguished.

For the description of the social interaction behavior, we classified the bat that was foraging continuously around a street light (at illuminated sites) or was consistently present within the range of our microphones (at unlit sites) as ‘resident’. A second bat that then entered the space was classified as ‘intruder’, since it entered a foraging site where the resident was already present.

### 3D-flight path reconstruction

By using microphone arrays for audio recordings, we were able to reconstruct the flight paths and subsequently the flight behavior of recorded bats. Calls of bats flying above the T-shaped array reached the four microphones at different times due to different distances between the emitter and the receivers. These differences in arrival time encoded the spatial positions of the bats in each moment of signal emission relative to the reference microphone, which was part of the array. Time-Of-Arrival-Differences (TOAD) were determined by measuring the time lag at which the cross-correlation function between the signal received by the reference microphone and the signals received by each of the other three microphones reached the maximum value. By using the three calculated TOADs we could determine the three-dimensional positions of the bats above the array in each moment of signal emission. The positions from consecutive calls of individual bats were then assembled and smoothed using a moving average filter for nine consecutive calls to estimate continuous individual flight paths, providing individual coordinates for each millisecond of the recording. We labeled different signal types (echolocation calls, Type 1 and Type 2 calls) with different colors for their discrimination in results. For the calculation of TOADs and the reconstruction of flight paths we used the software Matlab R2016a (Math Works, Massachusetts, USA) and designed appropriate programs.

**Figure 1:**
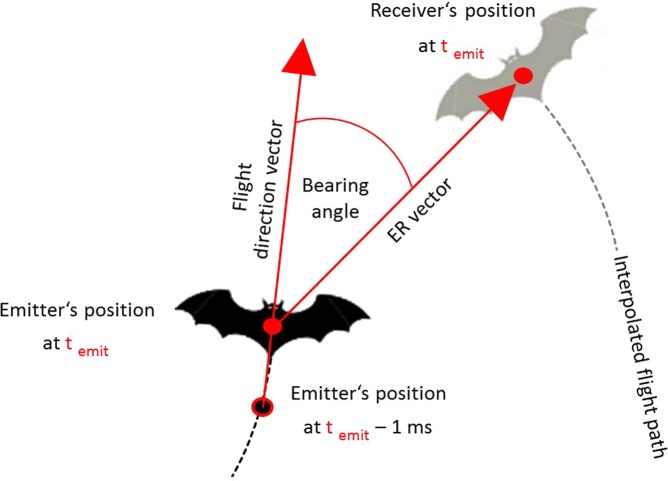
Determination of bearing angle and inter-individual distance between emitter and receiver. The bearing angle was measured between the flight direction vector of the emitter (black bat) and the ER vector between emitter and receiver (grey bat) in the moment of call emission (t_emit_). The flight direction vector was defined by the spatial position of the emitter at t_emit_ and its interpolated spatial position 1 ms earlier.

We compared the spatial relations between bats during emissions of Type 1 calls and Type 2 calls. For the calculation of the bearing angles between a bat emitting a Type 1 or Type 2 call and the bat receiving the Type 1 or Type 2 call, we used the spatial position of the emitter in the moment of signal emission and its interpolated spatial position 1 ms before the call emission to create a flight direction vector. This vector was set to be the flight direction with an angle of 0°. The spatial position of the bat receiving the call was taken from its interpolated flight path at the point of time the call was emitted. The bearing angle was measured between the flight direction vector and the vector from the emitter’s position to the receiver’s position at call emission (ER vector, Fig. 1). Differences in inter-individual angles for Type 1 and Type 2 call emissions were compared by an ANOVA (JMP 11.2.0). We also determined the inter-individual distance between the two bats in the moment of Type 1/Type 2 call emission. Distances were not normally distributed and log-transformed before conducting an ANOVA (JMP 11.2.0).

## Results

### Type 1 and Type 2 call emissions by *Pipistrellus pipistrellus*

**Figure 2:**
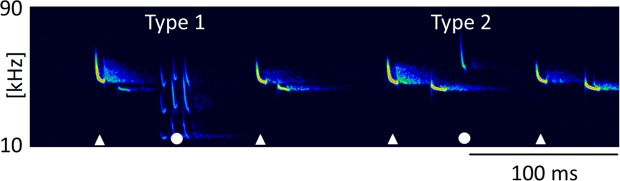
Type 1 and Type 2 call between echolocation signals. Type 1 call and Type 2 call emitted by a resident in mutual flight with an intruder. Calls of the resident are marked by white symbols, with Type 1 and 2 calls (circles) interspersed between search calls (triangles).

We reconstructed flight paths of 12 audio recordings of foraging residents and intruders of *P. pipistrellus* containing 60 Type 1 calls and 74 Type 2 calls. In 11 of 12 recordings with Type 2 calls, both call types were present, whereas in one recording Type 1 did not accompany a Type 2 call. In 8 of 12 recordings, Type 1 calls were emitted shortly before Type 2 calls, with a median interval from beginning of Type 1 call to beginning of Type 2 call of 510 ms (Q1: 310 ms, Q3: 930 ms, n = 20, Fig. 2). Across all recordings, Type 1 and 2 were emitted exclusively by the resident with the exception of one case, where an intruder also emitted a Type 1 call.

### Type 1 calls

Type 1 calls of *P. pipistrellus* consisted of 3–5 multiharmonic and frequency-modulated syllables with terminal frequencies of 16.0 ± 0.8 kHz (mean ± SD, n = 50). These complex calls lasted on average 25 ms (25.0 ± 3.9 ms, mean ± SD, n = 50) and were interspersed between echolocation signals. The number of echolocation calls between Type 1 calls varied from 2 to 83 signals within discrete recording sequences of 20-second duration. The social calls were emitted during foraging flight, indicated by feeding buzzes and typical pursuit behavior.

### Type 2 calls

**Figure 3:**
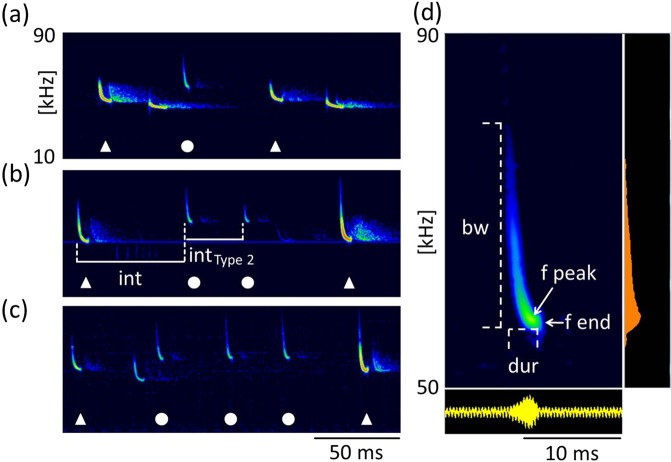
Type 2 calls. Type 2 calls were emitted by the resident in presence of an intruder as single calls (**a**) or in groups of two (**b**) or three (**c**) consecutive calls. Calls of the resident are marked by white symbols, with Type 2 calls (circles) interspersed between search calls (triangles). Call parameters for characterization of Type 2 calls are bandwidth (bw), duration (dur), peak frequency (f peak) and terminal frequency (f end), (**d**). Pulse intervals of Type 2 calls were measured to the preceding search call (int, b) and within grouped Type 2 calls (int Type 2, b).

**Figure 4:**
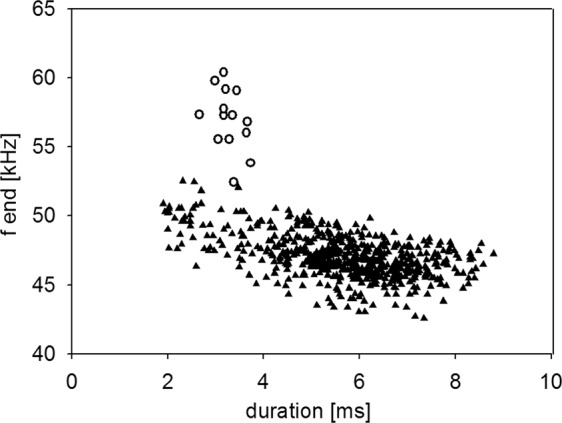
Correlation of terminal frequency (f end) of Type 2 calls and search call*s* with call duration. Type 2 calls (white circles) have higher terminal frequencies than search calls (black triangles).

Type 2 calls were short, frequency-modulated calls with terminal frequencies above the terminal frequencies of echolocation signals of the emitter. They were interspersed between echolocation signals and delivered as either single calls, or groups of two or three consecutive calls (Fig. 3a–c). These calls were emitted by the resident and were recorded exclusively in presence of an intruder. A single recording revealed an emission in presence of an interspecific intruder (sp. *Pipistrellus nathusii*). The terminal frequency of Type 2 calls ranged from 51.3 to 60.4 kHz (56.7 ± 2.1 kHz, mean ± SD, n = 27) and was 4.5 to 13.9 kHz (8.8 ± 2.2 kHz mean ± SD, n = 27) higher than the terminal frequency of the preceding echolocation call by the same individual. Analysis of terminal frequency and duration for all Type 2 calls and search calls that fulfill the criterion of sufficient amplitude revealed significantly higher terminal frequencies (57.0 ± 2.2 kHz, Mean ± SD, n = 14) for Type 2 calls than search calls (48.7 ± 1.5 kHz, Mean ± SD, n = 35, Anova, F(1,47)=232.13, p < 0.0001) of comparable duration (2.7–3.7 ms, Fig. 4). Type 2 calls had peak frequencies of 58 ± 2.4 kHz (mean ± SD, n = 73), bandwidths of 6.4 ± 1.7 kHz (mean ± SD, n = 14) and durations of 3.3 ± 0.3 ms (mean ± SD, n = 14, Table 1).

**Table 1:** Parameters of Type 2 calls.

	duration [ms]	f peak [kHz]	bandwidth [kHz]	f end [kHz]
n	14	73	14	26
mean	3.3	58.0	6.4	56.7
SD	0.3	2.4	1.7	2.2
min	2.7	52.1	4.2	51.3
max	3.7	64.3	9.3	60.4

### Pulse intervals

In search flight, *P. pipistrellus* emitted echolocation signals with durations of up to 10 ms and pulse intervals around 95 ms (Median 95.4 ms, Q1: 80.8 ms, Q3: 107.6 ms, n = 2355). Pulse intervals of ~90 ms indicate sound emissions most likely in rhythm with wing beat, whereas a pulse interval of ~180 ms indicates a wing beat without sound emission.

Type 1 and Type 2 calls were interspersed between echolocation signals and their emission affected the pulse intervals of echolocation calls in the following way:

The median pulse interval from the beginning of Type 1 calls to the beginning of the preceding echolocation call was 46.8 ms (Q1: 44.2 ms, Q3: 51.4 ms, n = 60) and from the beginning of Type 1 calls to the beginning of the following echolocation call was 85.2 ms (median, Q1: 76.8 ms, Q3: 102.8 ms, n = 58). Resulting from the insertion of a Type 1 call, the pulse intervals between echolocation signals increased to median values of 133.6 ms (Q1: 124.2 ms, Q3: 154.2 ms; n = 59, Wilcoxon, p < 0.0001).

Type 2 calls were interspersed with median intervals of 53.5 ms (Q1: 45.8 ms, Q3: 60.1 ms, n = 58) to the preceding echolocation call. However, in contrast to Type 1, the interspersion of Type 2 calls did not always affect the pulse intervals of echolocation signals. Single interspersed Type 2 calls induced no extension of pulse intervals between echolocation signals (median 96.9 ms, Q1: 89.4 ms, Q3: 121.5 ms, n = 43, Wilcoxon, p = 0.0572), groups of two Type 2 calls induced extended pulse intervals between echolocation calls of 152.1 ms (median, Q1: 144.9 ms, Q3: 162.5 ms, n = 12, Wilcoxon, p < 0.0001) and a group of three consecutive Type 2 calls induced a median pulse interval of 169.4 ms between the preceding and following echolocation call of the emitter.

Pulse intervals of grouped Type 2 calls lasted 34.2 ms (median, Q1: 29.2 ms, Q3: 39.3 ms, n = 14).

### Flight behavior

#### Type 1 calls

**Figure 5:**
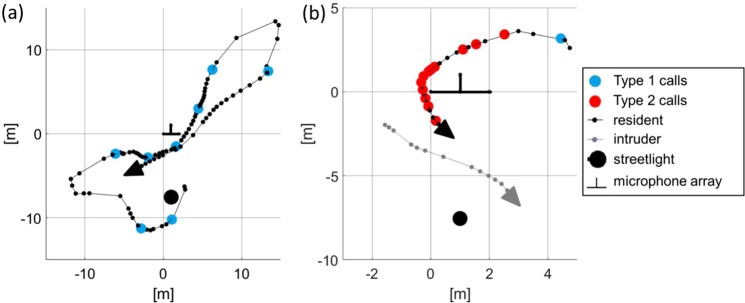
Top view of the flight paths of a foraging resident in single flight (**a**) and in flight with an intruder (**b**). Small dots indicate positions where echolocation signals were emitted by the resident (black) or by the intruder (grey). Large dots indicate Type 1 and Type 2 signals. Type 2 calls (red dots) were emitted exclusively by the resident in presence of an intruder, often during chase flights.

Type 1 calls were emitted by the resident in single flight and in flights with an intruder (Fig. 5a,b). Flight path reconstructions revealed flights along constant routes, interrupted by turning patterns with feeding buzzes, as usually observed in foraging bats. No false landings or gliding elements such as those reported from songflights were observed. The resident often emitted Type 1 calls shortly before a conspecific (or occasionally an individual of a different species) intruder emerged.

#### Type 2 calls

**Figure 6:**
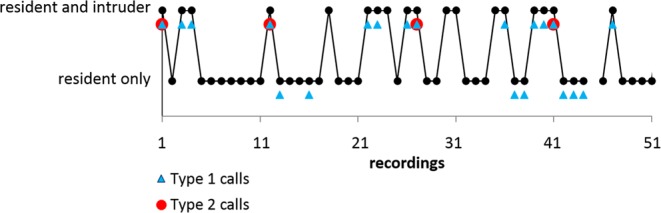
Emission pattern and effect of Type 2 calls. Example of vocal behavior of a resident individual foraging alone or in the presence of intruders documented by 50 consecutive recordings each with a duration of 20 seconds. Recordings are interrupted by approx. 0.9 seconds for data saving by the recording device (total evaluation time 18 minutes). Black dots indicate that the resident only or both resident and intruder were present as evidenced by their echolocation signals. Recordings where the resident emitted one or more Type 1 calls are marked by blue triangles, recordings with Type 2 calls are indicated by red circles. The resident emitted Type 2 calls exclusively in presence of intruders. After the emission of Type 2 calls intruders left the foraging site and were not traceable during the subsequent audio recording.

Type 2 calls were emitted only by the resident and exclusively in the presence of an intruder (Fig. 5b). Flight paths of residents and intruders revealed that Type 2 call emission mostly took place either during chases of the intruder by the resident (60%) or in frontal encounters of both bats (25%). In both flight situations the resident flew directly towards the intruder. Few Type 2 calls were emitted when flight paths already crossed (15%) and were still in close proximity. Analysis of consecutively-taken audio recordings revealed that intruders left the recording area after receiving Type 2 calls from the resident (Fig. 6).

### Bearing angle and inter-individual distance between resident and intruder at Type 1 and Type 2 call emission

**Figure 7:**
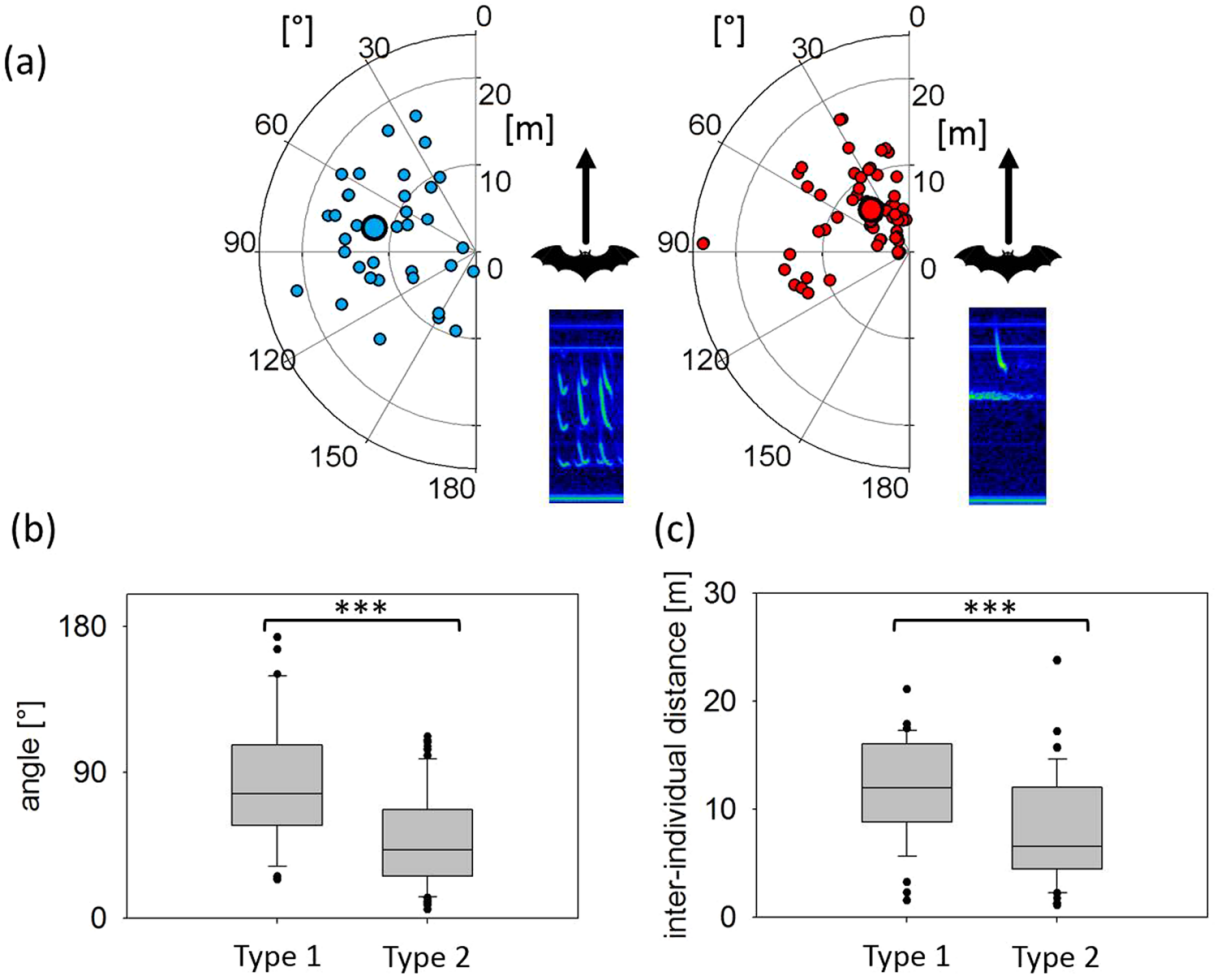
Spatial positions of intruders in relation to the position and flight direction of the resident at the emission of Type 1 and Type 2 calls. Spatial positions of intruders (small circles) at Type 1 call (left) and Type 2 call emission (right) by the resident with median position (large circles) indicated by distance and angle relative to the resident’s position and flight direction (0°) (**a**). Comparison of relative angles (**b**) and distances (**c**) at the emission of Type 1 (n = 36) and Type 2 calls (n = 68) revealed significant differences. Bearing angles and inter-individual distances were smaller at Type 2 call emissions than for Type 1 call emissions.

During Type 1 call emission, the bearing angles to the intruder varied widely, with a range from 23.9 to 173.5°. The values for the same measurement at Type 2 call emission were less variable and ranged from 5.3 to 112.2°. Bearing angles to the intruder were significantly smaller during Type 2 call emissions (42.1 ± 3.6°, median ± SEM, n = 68) than during Type 1 call emissions (76.8 ± 6.5°, median ± SEM, n = 36; Anova, F(1,102) = 26.82, p < 0.0001, Fig. 7a,b). Inter-individual distances between resident and intruder were also significantly smaller during Type 2 call emissions (6.5 ± 0.6 m, median ± SEM, 1.1–23.8 m, min-max, n = 68) than during Type 1 call emissions (12.0 ± 0.8 m, median ± SEM, 1.5–21.1 m, min-max, n = 36; Anova, F(1,102)=11.74, p = 0.0009, Fig. 7a,c).

### Annual distribution of social calls

Type 1 and Type 2 calls were recorded throughout the entire activity season from May to October (Table 2). As shown by the annual distribution, Type 1 call emission was evenly distributed over 82% of all recording nights, whereas Type 2 was emitted in 45% of nightly samples and occurred only in detached nights in July, August and September. The highest rates of Type 1 and Type 2 calls were recorded in October.

**Table 2:** Annual distribution of Type 1 and Type 2 call emissions.

month	n Type 1 calls / hour	n Type 2 calls / hour	recording hours	n recording nights	n recording nights with Type 1 calls	n recording nights with Type 2 calls
May	25.2	37.6	4.6	5	4	3
Jun	15.5	1.8	7.6	4	4	2
Jul	44.1	43.7	6.2	4	3	1
Aug	18.9	6.8	8.1	4	3	1
Sep	31.7	3.7	5.7	3	2	1
Oct	236.5	77.6	5.5	2	2	2

## Discussion

Our sound recordings with an array of four microphones allowed the reconstruction of the three-dimensional flight paths of foraging pipistrelles flying either alone or together with conspecifics and the calculation of the bats’ positions at the emission of the recorded sounds. With this method we are able to attribute the emission of specific calls to particular behavioral situations. In addition to echolocation signals, we recorded two other types of calls from foraging common pipistrelles. One type, termed “Type 1 call”, was described in former publications and identified as social call^2,11,12,15–17^. The other type, termed “Type 2 call”, had yet to be described, but we hypothesize based on the study presented here that it too serves a social function (see below).

The social call “Type 1” consists of 2–5 multi-harmonic frequency-modulated syllables and was named “complex social call” by Pfalzer and Kusch (2003). We will adopt this term for the discussion of our data. Complex social calls have been found in sound recordings from foraging bats but also in recordings from pipistrelle males while performing so-called “songflights” in their mating territory^10^. Sound sequences consisting of echolocation signals with interspersed complex social calls have been named flightsongs by Smotherman at al. 2006, and both foraging and courtship flightsongs^3,10,11,14,15,17,24^ have been interpreted as a notable example of territorial singing^3,10,12,13,16,25,26^. Many studies have attempted to identify functional differentiation between complex social calls emitted during courtship and foraging, e.g. by naming them advertisement or agonistic calls^2,9–13,24,27,28^. Arguments for this differentiation are based on observations that complex social calls attracted females to mating grounds but were also observed in courtship and foraging flightsongs emitted at sites where chasing behavior also occurred. Our data appears to resolve this apparent discrepancy at least for foraging *Pipistrellus pipistrellus*, by attributing the recorded signal types to specific behavioral situations.

Flightsongs were emitted by foraging residents independently from the presence or absence of conspecifics. However, in situations where we only recorded the resident, this individual may have sensed the echolocation calls of an approaching intruder and reacted with social call emission before our recording equipment picked up the conspecific’s signals. In situations with two bats, the reconstructed flight paths of both individuals revealed that exclusively the resident emitted complex social calls. Only once, an approaching intruder emitted a complex social call, while the resident oscillated between two street lamps. We assume this was an attempt to occupy the territory around the streetlight while the resident was at the neighboring lamp. In this case, however, the resident returned and repelled the intruder. Complex social calls were always emitted before chasing the conspecific away from the food source. In contrast to reports from former studies, we show that the complex social calls were emitted only prior and not during chasings and therefore do not support an agonistic function. If a conspecific was traceable, the inter-individual distance and the bearing angle between resident and intruder were rather high, revealing an undirected broadcast of the signal. This supports our hypothesis that complex social calls serve as a general announcement of the emitter’s presence and display territorial behavior. Our findings here support the conclusions of former studies, that bats emit complex social calls as a warning to intruders^11,29^ and that foraging flightsongs have a territorial character^3,30^.

If these warnings were disregarded by intruders and conspecifics entered the claimed territory, the resident reacted with agonistic behavior and chased these individuals away from the food source. During these chases the resident emitted Type 2 calls, which we term “high frequency social calls” based on their frequency structure. Single or groups of high frequency social calls were interspersed between echolocation signals and were emitted at short distances between intruder and resident and at minor bearing angles, indicating they were specifically addressed to the intruder. Their reduced range caused by increased sensitivity to atmospheric attenuation due to their high frequency also supports this conclusion. Both behaviors, chasings in combination with high frequency social call emission, preceded the intruder’s departure. This supports the hypothesis that high frequency social calls have a repelling effect on the behavior of conspecifics and are true agonistic social calls.

The attribution of complex and high frequency social calls to specific behavioral situations showed that the occupation and defense of a food source is a two-stage process: A resident produces foraging flightsongs with complex social calls to claim a resource and to warn conspecifics of entering its territory (claiming phase). The disregard of this warning leads to an escalation where the resident defends the food source by reacting to the intruder with chasings and accompanied high frequency social calls (agonistic phase).

Flightsongs can also occur as part of courtship behavior, when bats lay claim to a mating territory^11–13,23^. It is not surprising, then, that complex social calls in foraging and courtship flightsongs do not essentially differ in structure, but allow the individual recognition of a resident by individualized signal design^2,12,16,31^. Complex social calls are likely to transmit information such as species, sex, age, weight and size of their sender and qualify other bats to estimate or even recognize the characteristics of the territory holder. This may enable female bats to choose a mating partner and help avoiding costly agonistic interactions between intruders and residents.

The incidence of flightsongs and high frequency social calls during the activity season of *P. pipistrellus* reflects phases of low food availability in spring and fall as well as the mating season in late summer. The number of complex social calls increases when insect density decreases^11,29,32,33^, with time after dusk^34,35^, or due to falling air temperatures^36^, hence when competition for food increases. Under such circumstances, the claiming of a food patch may be necessary for residents to avoid potential agonistic interactions with other bats. High frequency social calls were recorded primarily in May and October, when weather conditions are poor and insect activity is considerably reduced. Since insects are scarce at these times of the year, bats might engage in riskier behaviors more often by intruding into already occupied territories and eliciting agonistic food source defense behaviors by the resident. According to our data, high frequency social calls also occurred more often in July, but only in one of four recording nights. In this case, a decrease in insect activity due to unfavorable weather conditions may have led to higher competition for food.

Our results suggest that high frequency social calls have an agonistic function and serve to repel other bats from food sources when competition for food is high. Our work suggests that bats use complex social calls for claiming resources such as courtship or foraging territories. However, these calls are not necessarily associated with agonistic behavior. Whether high frequency social calls are also emitted by males for the defense of their mating territories requires further investigation.

## Data Availability

The datasets generated during and/or analyzed as part of the current study are available from the corresponding author upon request.
